# Health technology assessment framework for artificial intelligence-based technologies

**DOI:** 10.1017/S0266462324000308

**Published:** 2024-11-21

**Authors:** Rossella Di Bidino, Signe Daugbjerg, Sara C. Papavero, Ira H. Haraldsen, Americo Cicchetti, Dario Sacchini

**Affiliations:** 1 Graduate School of Health Economics and Management, Universita Cattolica del SacroCuore (ALTEMS), 00168 Rome, Italy; 2 Departement of Health Technologies and Innovation, Fondazione Policlinico Universitario Agostino Gemelli IRCCS, 00168 Rome, Italy; 3 Department of Neurology, Division of Clinical Neuroscience, Oslo University Hospital, Norway; 4 Directorate-General for Health Programming, Ministry of Health, Italy; 5 Fondazione Policlinico Universitario Agostino Gemelli IRCCS, 00168 Rome, Italy; 6 Department of Healthcare Surveillance and Bioethics, Universita Cattolica del Sacro Cuore, 00168 Rome, Italy

**Keywords:** artificial intelligence, health technology assessment, value assessment, AI-HTA framework, AI-Mind Study

## Abstract

**Objectives:**

Artificial intelligence (AI)-based health technologies (AIHTs) have already been applied in clinical practice. However, there is currently no standardized framework for evaluating them based on the principles of health technology assessment (HTA).

**Methods:**

A two-round Delphi survey was distributed to a panel of experts to determine the significance of incorporating topics outlined in the EUnetHTA Core Model and twenty additional ones identified through literature reviews. Each panelist assigned scores to each topic. Topics were categorized as critical to include (scores 7–9), important but not critical (scores 4–6), and not important (scores 1–3). A 70 percent cutoff was used to determine high agreement.

**Results:**

Our panel of 46 experts indicated that 48 out of the 65 proposed topics are critical and should be included in an HTA framework for AIHTs. Among the ten most crucial topics, the following emerged: accuracy of the AI model (97.78 percent), patient safety (95.65 percent), benefit–harm balance evaluated from an ethical standpoint (95.56 percent), and bias in data (91.30 percent). Importantly, our findings highlight that the Core Model is insufficient in capturing all relevant topics for AI-based technologies, as 14 out of the additional 20 topics were identified as crucial.

**Conclusion:**

It is imperative to determine the level of agreement on AI-relevant HTA topics to establish a robust assessment framework. This framework will play a foundational role in evaluating AI tools for the early diagnosis of dementia, which is the focus of the European project AI-Mind currently being developed.

## Introduction

Recent advancements in artificial intelligence (AI) have demonstrated successful results in various clinical practices, and there is increasing anticipation of AI-based technologies addressing the global healthcare crisis. This crisis arises from a shortage of healthcare professionals, aging populations (1), and limited financial resources (2).

AI encompasses a wide range of applications and technologies. As defined by the Organisation for Economic Co-operation and Development (OECD), AI is “a machine-based system that, for explicit or implicit objectives, infers, from the input it receives, how to generate outputs such as predictions, content, recommendations, or decisions that [can] influence physical or virtual environments. Different AI systems vary in their levels of autonomy and adaptiveness after deployment” (3;4;5).

In the context of health, AI-based technologies serve as an umbrella term, including machine learning algorithms and other cognitive technologies that utilize medical data to automate specific tasks. These applications aim to cover the entire patient journey, supporting clinicians in diagnosis, therapeutic decision making, and predictions (6).

Globally, the healthcare market size of AI is estimated to be USD 15.4 billion in 2022, with an expected compound annual growth rate of 37.5 percent from 2023 (7). In Europe, as well as in other parts of the world, health has been recognized as a key application for AI. Nevertheless, a significant need for AI regulatory frameworks and a code of practice to address healthcare-specific risks and requirements has been highlighted. This recognition is evident in various documents, including the European Strategy on AI from 2018 (8), the Guidelines for Trustworthy AI in 2019 by the High-Level Expert Group on AI (9), and the recently proposed legal framework on AI, which is the first of its kind, introduced in April 2021 (10). Notably, the European Parliament has recently released the first regulation on AI (11).

The implementation of new medical technologies and clinical pathways in health care is firmly grounded in research and an evidence-based scientific approach. It is a standard practice for clinicians to rely on health technology assessment (HTA) methods to aid in the decision-making process regarding the adoption of new technologies (12). The purpose of HTA is to support and inform policy decision making based on a systematic and evidence-based approach. In Europe, the primary reference framework for HTA is the European Network for HTA (EUnetHTA) Core Model (13), which guides assessors through a comprehensive evaluation of technologies across nine different domains. This model has been developed and adopted for medical devices and pharmaceutical products, enjoying broad recognition of its value among relevant stakeholders including industry (14;15). However, AI-based health technologies (AIHTs) challenge the applicability of traditional HTA methods due to innovative technologies evolving at a pace faster than the methods used for conducting HTA. Key challenges posed by AIHTs from the HTA perspective include the following:

- Nature of AI (16), given that these types of technologies may (as is the case with adaptive algorithms) or may not continue to evolve.
- Lack of transparency and replicability (17).
- Ethical and legal implications are widely debated both in a general context (18) and concerning specific clinical applications, such as breast cancer (19), among others.

Furthermore, the limitations or inability of most AI technologies to “explain” their decision-making process underscores the importance of updating traditional HTA methods. This update should include new aspects such as trustworthiness (20;21), transparency, interpretability (22), and explainability (23;24) within the HTA framework. These additions are crucial to provide decision makers with the proper support when considering the adoption of AI (25).

Despite attempts to align HTA methods with AI adaptation, such as the Model for ASsessing the value of AI in Medical Imaging (MAS-AI) (26), Digi-HTA in Finland (27;28), AQuAS Framework for digital health (29), and the evidence standards framework adopted by NICE for digital health technologies (30), no joint agreement among experts exists on how to assess the value and effect of AI-based technologies.

Therefore, the objective of this study is to examine the perception among European healthcare decision makers, assessors, and experts regarding the usability of the EUnetHTA Core Model for assessing AI-based technologies. Additionally, the study aims to explore their perception of including new assessment topics identified in the literature as important for the assessment of AI-based technologies.

This study is being conducted as part of the European Union (EU) project “The AI-Mind” (31), supported by the European Research and Innovation Action Plan (No. 964220). The main objective of the EU project is to develop AI-based diagnostic tools for early screening and risk assessment to predict the onset of dementia (32). Subsequently, the study aims to evaluate the usability of the developed tool (33;34). In the absence of HTA frameworks that support the evaluation of AI-based technologies, it has been decided, as a first step, to initiate an early dialog among stakeholders. This dialog will involve patients, developers, industry representatives, clinicians, and HTA experts, with the purpose of setting priorities and identifying the evidence required to inform decision-making processes.

## Methods

Our analysis was conducted in three steps. First, a list of potential HTA topics relevant to AI was identified based on a scoping review. Subsequently, a Delphi survey was conducted among qualified experts, and our approach adhered to the guidelines for the Delphi survey (35;36).

### Step 1: Identification of HTA Issues Relevant for AI

An initial catalog of HTA domains and topics was created based on the EUnetHTA Core Model (version 3.0) (13). Following this, a rapid review was conducted to identify additional relevant topics for inclusion. This involved searching both scientific and gray literature across platforms such as PubMed, Cochrane Library, Google Scholar, and websites of major HTA agencies. Additionally, we considered abstracts from HTAi annual meetings, the ISPOR Presentations Database, and INAHTA member resources.

The syntax used for the literature review in PubMed is provided in Supplementary File 1a, and the websites of the HTA agencies included in our analysis are listed in Supplementary File 1b. Furthermore, we identified reports covering AI topics in health from international institutions such as the World Health Organization (WHO), the European Commission, and the OECD.

### Step 2: Delphi Survey

Topics identified in Step 1 were used in the development of the Delphi survey. A modified version of the Expert Delphi technique was selected for consensus building, and it involved the following four steps: (i) Development of an online survey. (ii) Recruitment and consenting of participants to the Delphi panel. (iii) Two rounds of consultation on the proposed topics in the survey. (iv) A webinar for the expert panel.

#### Design

The survey covered the nine domains and associated topics presented in the EUnetHTA Core Model and additional topics identified through the rapid review. In total, the survey comprised sixty-five multiple-choice questions. Each topic was briefly described, and after each domain, a free-text question was included for the panelists to provide comments. The full list of domains and related topics is reported in Supplementary File 2.

Information regarding occupation, expertise, knowledge about HTA and AI, and geographical location was also collected from each panelist.

For the consensus process of the Delphi survey, panelists used a 9-point Likert scale to rate each statement. A score of 9 indicated the highest level of agreement for inclusion, whereas a score of 1 suggested that the topic should not be included in an HTA of AI-supported technologies. The survey specified that a score from 1 to 3 should be interpreted as “should not be included in an HTA on AI,” a score from 4 to 6 as “important but not critical to include in HTA of AI,” and a score higher than 7 as “critical to include in HTA of AI.”

The electronic survey was prepared using the user-friendly Alchemer online interface (www.alchemer.com).

We used 70 percent as a cutoff for high agreement among experts for each topic. This cutoff was applied not at a single point on the Likert scale but for each of the three categories described above (critical to include, important not critical, and not important).

The choice of the cutoff was based on guidelines (37) and works from the WHO (38;39;40). For example, if ≥70 percent of the responses fell in the range of seven to nine, the topic was considered critical and to be included in the assessment of AIHTs.

#### Participants

Our goal was to assemble a multidisciplinary expert panel.

Potential participants for the Delphi panel were identified based on their publications, CVs, area, and level of expertise. In terms of geographical representativeness, the primary focus was on Europe-based experts. We identified nine relevant categories of experts: (i) clinician/researcher; (ii) HTA; (iii) technical experts (e.g., data programmer/engineer and cybersecurity); (iv) ethicist/bioethicist; (v) patients/advocates; (vi) health economy; (vii) health policy; (viii) legal aspects; (ix) user experience. To ensure representation from all categories in the Delphi process, we aimed to have a minimum of five representatives from each main expert group (groups 1–2–3-6) participating in the survey. Anticipating a response rate in the range of 30–40 percent, a total of 87 experts were invited.

#### Data Collection

Experts were invited to participate in the Delphi survey through email, which outlined their expected involvement and rights as participants, along with a link to the online survey. We conducted a two-round online Delphi survey. The second round duplicated the questions from the first round, but participants could view ratings (percentage of respondents according to importance score) from the initial round. This allowed them to adjust, confirm, or reconsider their answers. Finally, the expert panel was invited to a webinar to discuss the results of the survey and provide additional feedback on the survey, AI technologies, and HTA models for AI.

### Step 3: Statistical Methods

The analysis was conducted using Microsoft Excel, with key indicators focusing on the proportion/percentage of respondents based on the importance score and category for inclusion/exclusion from the HTA–AI framework.

In this paper, we present results obtained at the conclusion of the second round of the Delphi survey, along with findings gathered from the discussions held during the virtual workshop.

## Results

The Delphi survey spanned from April 2022 to January 2023, with the final webinar held in May 2023. Of the 87 experts invited, 46 responded to both rounds of the Delphi survey (Supplementary File 1c), resulting in a response rate of 47.4 percent, exceeding the anticipated value.

**Figure 1.**
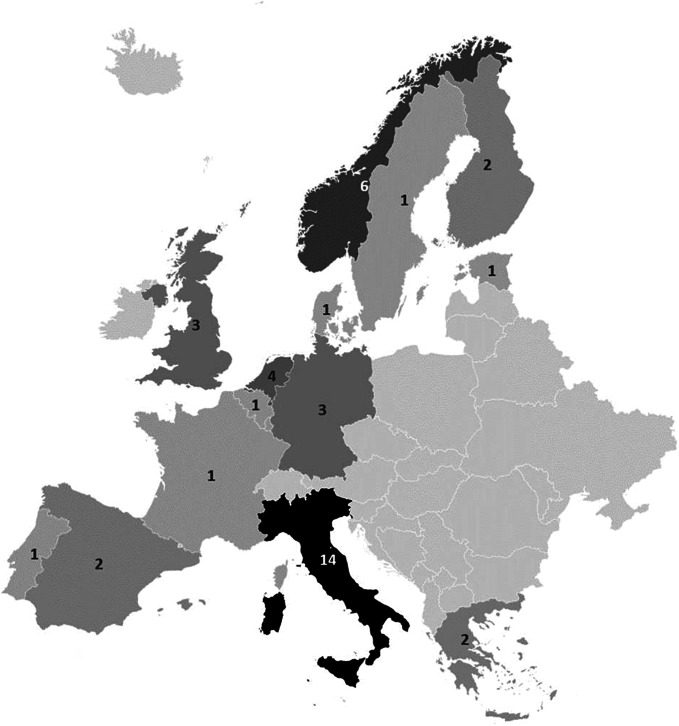
European Union countries represented in the panel.

The majority of respondents (*n* = 43, 93.5 percent) were from Europe, representing fourteen different European countries. Italy (*n* = 14, 30.4 percent) and Norway (*n* = 6, 13 percent) demonstrated particularly high response rates (see Figure 1). Additionally, three panelists were based in other continents, specifically Canada, Tunisia, and Australia.

As outlined in Table 1, 12 panelists (26.1 percent) were clinicians and 7 (15.2 percent) were HTA experts. The majority of panelists reported having prior knowledge of HTA (*n* = 37, 80.4 percent) and familiarity with the EUnetHTA Core Model (*n* = 28, 60.9 percent). A smaller number of panelists (*n* = 5, 10.9 percent) reported having competencies in all HTA domains. Twenty-one (45.7 percent) possessed expertise in the assessment of clinical effectiveness, and 20 (43.5 percent) had experience in costs and economic evaluation. Regarding the ethical, legal, and social implications domains, only three experts had experience in assessing legal aspects, whereas 16 (34.8 percent) had experience in the other two domains, as well as in evaluating the organizational impact of health technologies.

**Table 1.** Panel composition and expertise

	*N*	%
Panel composition		
Clinicians	12	26.1
Health technology assessment expert	7	15.2
Technical experts (i.e., engineer and cybersecurity)	6	13.0
Health economics	6	13.0
Patients/advocates	4	8.7
Ethicist/bioethicist	4	8.7
Health policy expert	3	6.5
Legal expert	3	6.5
User experience	1	2.2
Health technology assessment domain of expertise (multiple responses)		
Description and technical characteristics of technology	14	30.4
Safety	8	17.4
Clinical effectiveness	21	45.7
Costs and economic evaluation	20	43.5
Ethical analysis	16	34.8
Organizational aspects	16	34.8
Patients and social aspects	16	34.8
Legal aspects	3	6.5
All domains	5	10.9
No expertise in health technology assessment	9	19.6
Level of familiarity with artificial intelligence		
Highly familiar	14	30.4
Moderately familiar	16	34.8
Somewhat familiar	12	26.1
Not familiar	4	8.7

Approximately 65.2 percent (*n* = 30) of panelists reported at least a moderate level of familiarity with AI. Additionally, sixteen responders had practical experience in assessing or implementing AI-supported health technologies/solutions (Table 1).

### Delphi Panel Results

**Figure 2.**
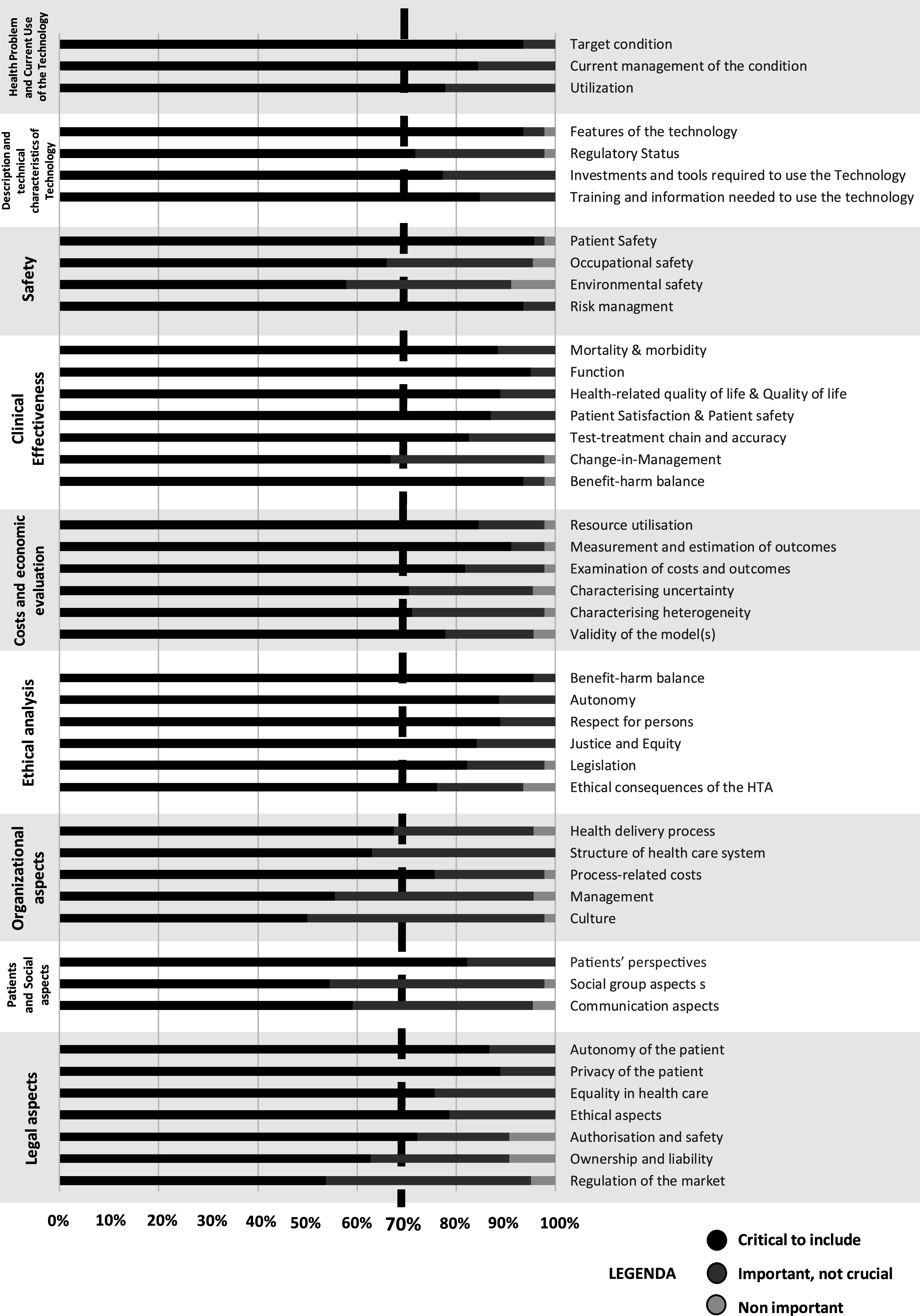
Summary of results for each traditional domain according to the EUnetHTA Core Model.

**Figure 3.**
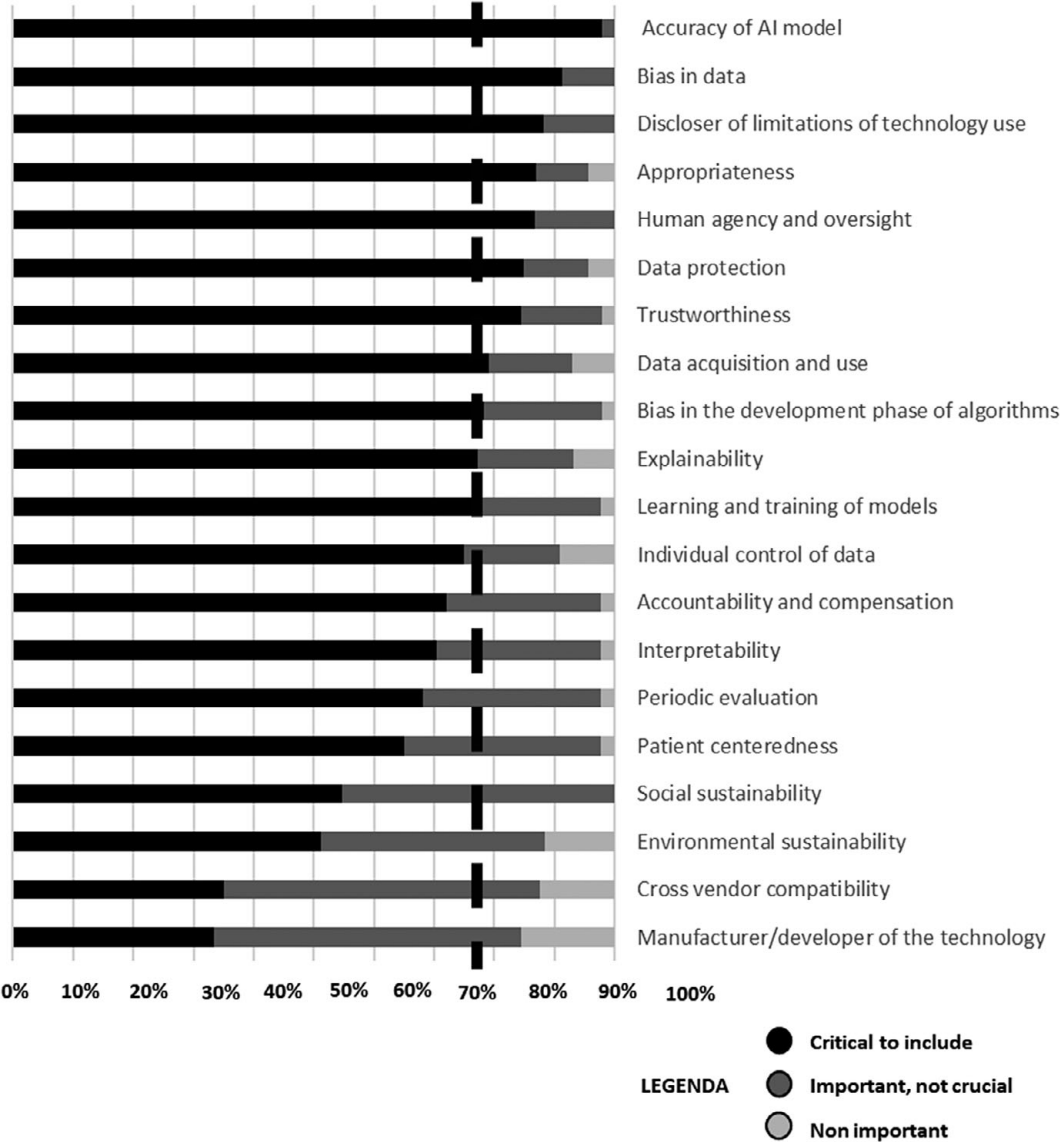
Summary of results for additional topics.

Overall, the Delphi panel expressed agreement on the importance of including 73.8 percent of the original topics suggested in the Core Model (refer to Figure 2) and 70 percent of the additional topics (refer to Figure 3) identified in the literature for the assessment of AI technology (deemed critical to include in HTA of AI). Further details for each topic are provided in Supplementary File 3.

Concerning the first domain, “health problem and current use of the technology,” our experts unanimously agreed on the inclusion of all topics in the HTA framework for AI. A similar consensus was reached for the four topics falling under the domain labeled “description and technical characteristics of technology,” although agreement on the Regulatory Status was close to the cutoff value, with 71.74 percent of panelists assigning a score of ≥7.

In the safety domain, our experts concurred on the inclusion of only two topics. Divergent perceptions were noted regarding the relevance of occupational and environmental safety. For clinical effectiveness, only one topic (“change-in-management”) fell into the exclusion area based on our criteria for assessing agreement.

Despite varying levels of agreement, all topics related to cost and economic evaluation emerged as crucial for inclusion in the framework. The lowest level of agreement was recorded for the characterization of uncertainty (70.45 percent) and heterogeneity (71.11 percent).

The importance of ethical aspects was evident from the collected responses, with no topics excluded. For organizational aspects, diverse scores were assigned, with agreement reached only for the cruciality of the topic “process-related costs” (75.56 percent). A similar pattern was observed for patient and social aspects, particularly for the topic of patients’ perspectives.

In the legal aspects, at least 70 percent of panelists considered 5 out of 7 topics crucial.

Figure 3 clearly shows how the proposed twenty additional topics captured crucial aspects of AI in the majority of cases (*n* = 14).

Upon reviewing the overall percentage of scores higher than seven, our panel identified the ten most crucial topics to incorporate into an HTA framework for AI-based technology. These topics are as follows: accuracy of AI model (97.78 percent), patient safety (95.65 percent), evaluation of benefit–harm balance from an ethical perspective (95.56 percent), function (95 percent), target condition (93.48 percent), technology features (93.48 percent), risk management (93.48 percent), evaluation of benefit–harm balance in the clinical effectiveness domain (93.48 percent), data bias (91.30 percent), and measurement and estimation of outcomes (91.11 percent).

**Figure 4.**
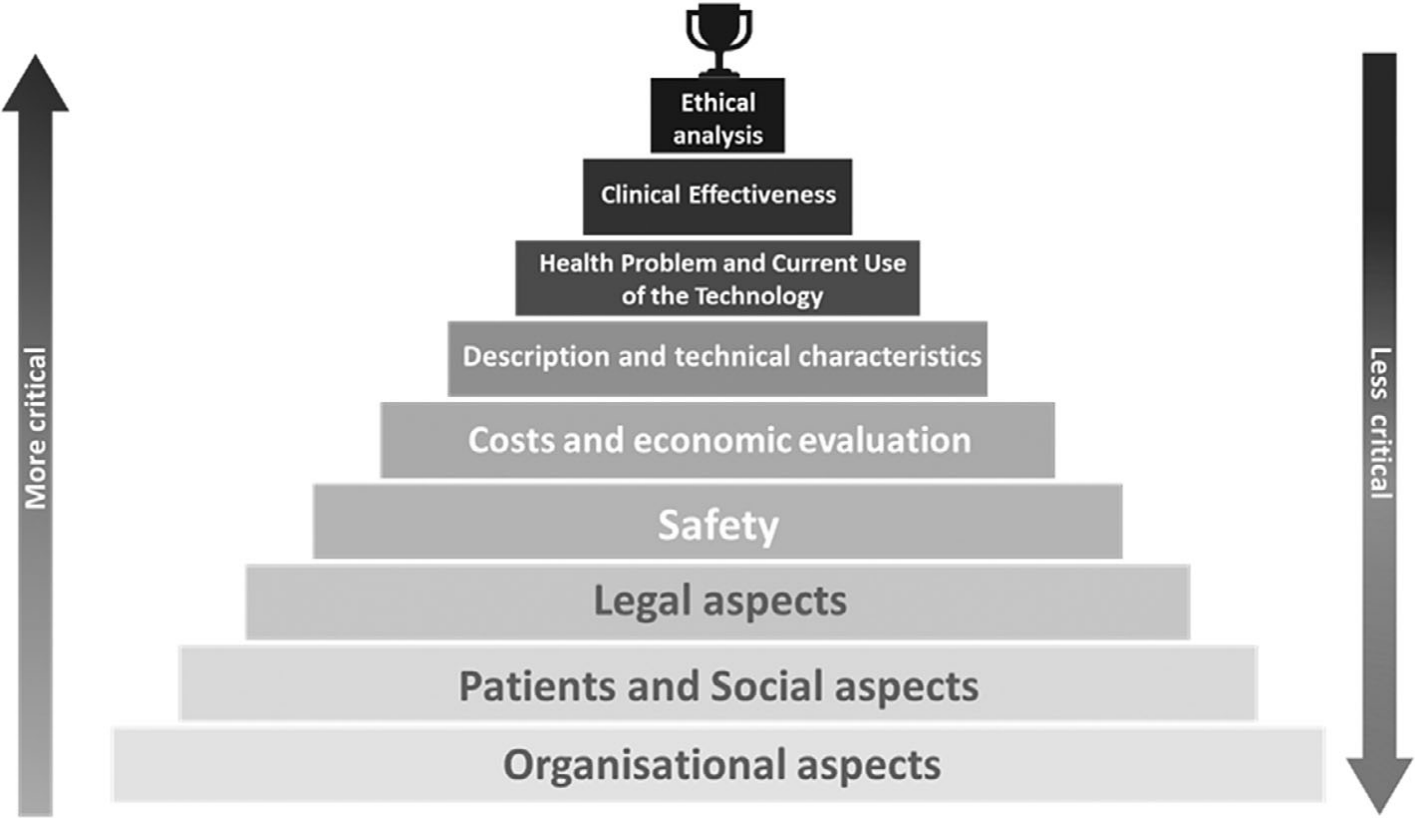
Hierarchy of health technology assessment domains as perceived by panel of experts.

By combining these findings with the proportion of topics to be included in the HTA framework for each domain, a ranking of domains was established, as depicted in Figure 4. Ethical analysis emerged as the most relevant domain, occupying the pinnacle of the pyramid, whereas organizational aspects appeared at the base as the least critical domain.

### Final Webinar

The results were communicated to all experts via email, and seventeen experts participated in the final webinar. During the 60-minute online meeting, feedback was collected regarding the results and the adopted methodology. First, the discussion underscored unanimous agreement among experts on the necessity for an AI-adapted HTA framework. It was emphasized that this framework should not be limited to the EUnetHTA Core Model but should instead serve as a flexible starting point. The recognition of the value of including additional topics was unanimous among panelists. Data-related topics—such as bias, acquisition, and application—are widely regarded as critica l in AI development, along with broader issues like human agency, oversight, and explainability.

The experts stressed the importance of the AI-adapted framework being flexible enough to accommodate the heterogeneity of AI technologies. They pointed out that AI encompasses a diverse array of technologies with applications ranging from primary/secondary prevention to diagnosis and patient management. It involves various end users such as clinicians, healthcare professionals, and patients. Thus, the relevance of certain topics may vary across different AI health technologies. The panel recognized the need to adapt the Core Model to specific types of technology and decision-making contexts, acknowledging the validity of the process that guided the survey’s definition.

However, the panel identified potential biases and limitations. Varying levels of expertise in HTA and AI among panel members could have influenced the results, impacting the interpretation of proposed topics. The lack of a detailed definition for each topic in the survey, particularly for Core Model topics, might have presented a “cultural” barrier for experts not aligned with the EUnetHTA evolution and tools. Conversely, experts in traditional HTA assessments might not have been familiar with new AI-related topics, such as learning and training of models. Additionally, the development of a specific HTA terminology for AI is still in progress, leading to potential differences in the interpretation of terms and topics like interpretability or trustworthiness.

Furthermore, some of the proposed topics are relatively new in HTA, and the inclusion of environmental impact assessment is particularly significant and challenging. Nevertheless, the environmental impact of AI has been already proved in terms of energy cost and related carbon emission. For instance, the carbon footprint of training a single big language model was estimated equal to around 300,000 kg of carbon dioxide emissions (41). Lack of experience and common methodologies may have contributed to the lack of agreement on this topic.

Finally, the results revealed some overlap among domains and topics in the EUnetHTA, as seen with the benefit–harm balance. However, it was acknowledged that topic overlapping is a well-known challenge of the Core Model and can be resolved during the adaptation phase.

## Discussion

Our analysis was based on a strong assumption – the widely recognized need for a dedicated framework for HTA in AI. We explored the specific levels of agreement or disagreement regarding the topics to be covered in an HTA–AI framework.

We presented an extensive list of potential relevant topics to a panel of experts in HTA and AI, including those listed in the EUnetHTA Core Model and additional ones. Through the Delphi survey, we were able to assess the level of agreement for each of these sixty-five topics, categorizing them into three groups: critical to include, important but not critical, and not important. By applying our criteria (≥70 percent of responses in the same group), the experts reached a consensus that forty-eight (73.8 percent) of the topics are critical and should be an integral part of an HTA framework for AI-based technologies.

Our findings highlighted the inadequacy of the Core Model in capturing all relevant topics for AI-based technologies. Experts unanimously supported the inclusion of 14 out of the 20 additional proposed topics in the HTA–AI framework. Interestingly, two of these topics – accuracy of the AI model and bias in data – were among the top ten most critical.

Furthermore, our analysis revealed the importance of ethical aspects in AI, placing them on the same level as, or even higher than, considerations of clinical effectiveness (see Figure 4). These results are consistent with other studies that identify new ethical, legal, and social challenges for the assessment of AI, with a focus on issues such as trust among clinicians and patients, as well as autonomy (16).

### Strengths

The study outlined in the paper distinguishes itself from other HTA frameworks proposed in the literature, ensuring that it is not a duplication. For example, while MAS–AI (26) shares a similar methodology – employing literature reviews and expert involvement – it is specifically tailored to medical imaging within the Danish context. In contrast, our perspective is more expansive, transcending limitations related to imaging or specific clinical indications. As highlighted in the paper, the Delphi panel, conducted as part of the AI-Mind EU project, intentionally avoided confining the research to dementia or Alzheimer’s disease. Despite a significant number of Italian experts (*n* = 14) in the final panel, our outreach spanned 15 EU and 3 non-EU countries (refer to Figure 1). Furthermore, our investigation into both the AI and HTA backgrounds of experts revealed that 65 percent of them were at least moderately familiar with AI (Table 1), and 80 percent had prior knowledge of HTA. These insights served to identify biases and limitations, guiding us toward potential areas of improvement. Notably, although there is a certain degree of overlap in results when comparing the MAS–AI domains (26) with our Figures 2 and 3, it underscores how some peculiarities of AI are universal across clinical applications. Looking forward, as the AI-Mind proposes an HTA–AI framework, experiences like MAS–AI will be invaluable in the adaptation process. During the final webinar, our experts affirmed the necessity for this adaptation, considering not only clinical applications but also expected end users and the technology readiness level (TRL) of the AI solution.

From the HTA perspective, the TRL (42) is significant in the assessment process (43). The AI-Mind platform is set to introduce two new AI-based tools: the AI-Mind Connector, which identifies dysfunctional brain networks through high-density electroencephalographic recordings, and the AI-Mind Predictor, which assesses dementia risk using data from the Connector. These data include advanced digital cognitive tests, genetic and protein biomarkers, as well as important textual variables. The overall objective is to deliver a medical device classified as 2b, with an expected achievement of TRL7 by the end of the project. It is important to note that the assessment process will begin before the complete development of the AI-based tools. This requires an interpretation of the results from our Delphi survey with a dual perspective – considering both early and comprehensive assessments. This nuanced approach aligns with the changing nature of AI development and emphasizes the need for adaptable HTA methodologies to suit evolving technological landscapes.

### Limitations and Developments

The authors acknowledge the research limitations at the current stage.

Regarding the Delphi survey, certain results may be influenced by the composition of the panel, which was not evenly distributed across areas of expertise, especially in the field of AI. Although we incorporated various perspectives, only a few patients/advocates chose to contribute to the study. To address this, the Patient Advocacy Lab of ALTEMS (Graduate School of Health Economics and Management) (44) will collaborate closely with the HTA group within the project to better capture patient perspectives. In the future, it should be considered the involvement of other categories of stakeholders (i.e., consumer and data protection organizations).

Additionally, no subgroup analysis has been conducted yet due to the limited sample size. We presented the list of candidate topics with a brief description (see Supplementary File 2) to our experts. Some topics relied on the EUnetHTA definition or interpretations found in studies. In other cases, we provided examples. Despite our efforts, it became evident before and after the survey that some topics lack validated and shared definitions, as is the case with explainability (24;45), interpretability (46), and trustworthiness (47). Similarly, certain results, such as the exclusion of environmental-related topics, may indicate a lack of experience in assessing the impact of the environment on health care rather than the lack of relevance of the matter. The evaluation of environmental consequences remains an evolving field in HTA (48;49;50) with unresolved issues (51).

Moreover, some results require further investigation, as seen in the low level of agreement on organizational impact (see Figure 4). This is particularly important because AI is anticipated to disrupt the organization of healthcare services (52;53).

### AI and HTA: Remit of the Study

The aim of our analysis was to contribute to the definition of an HTA–AI framework, rather than investigate the availability of evidence required by that framework. Previous studies, such as Farah (25) and Di Bidino **(54)**, have shown that current AI studies are not enough to meet HTA requirements. These studies have emphasized the need to improve evidence collection and HTA processes to adequately address the unique characteristics of AIHTs. Furthermore, our work does not examine the requirements and implications at the regulatory level. Lastly, the relevance of how AI-driven evidence could support assessments (55;56) is beyond the scope of our analysis.

## Conclusions

The development of an HTA framework should not only consider the characteristics of the specific category of technologies but also reflect the level of agreement among experts regarding what to assess. To facilitate the identification of an HTA framework for AI, a Delphi survey was conducted, involving 46 experts who selected 48 topics out of the 65 proposed. Not all of these topics are currently included in the EUnetHTA Core Model. The feedback collected from experts will play a crucial role in both defining the HTA framework and testing it with AI-based tools currently under development in the EU project AI-Mind. This project aims to support the early identification of dementia in patients with mild cognitive impairment.

## Supporting information

Di Bidino et al. supplementary material 1 Di Bidino et al. supplementary material

Di Bidino et al. supplementary material 2 Di Bidino et al. supplementary material

Di Bidino et al. supplementary material 3 Di Bidino et al. supplementary material

## Supplementary material

The supplementary material for this article can be found at http://doi.org/10.1017/S0266462324000308.
