# Supplementary material for: Health technology assessment framework for artificial intelligence-based technologies
Source: Int J Technol Assess Health Care. 2024 Nov 21;40(1):e61. doi: 10.1017/S0266462324000308 (PMC11703629; doi:10.1017/S0266462324000308)
Supplement: Di Bidino et al. supplementary material 1 — Di Bidino et al. supplementary material [file S0266462324000308sup001.docx]

## **Supplementary Material 1**

## **1a – Pubmed string**

("technology assessment*"[Title/Abstract] OR "technology overview*"[Title/Abstract] OR "technology assessment, biomedical"[MeSH Terms] OR "HTA"[Title/Abstract] OR "HTAs"[Title/Abstract]) AND ("telemed*"[All Fields] OR ("remote consultation"[MeSH Terms] OR ("remote"[All Fields] AND "consultation"[All Fields]) OR "remote consultation"[All Fields]) OR (("distance"[All Fields] OR "distances"[All Fields]) AND "Conseling"[All Fields]) OR ("econsult*"[All Fields] OR "e consult*"[All Fields] OR "ecounsel*"[All Fields] OR "e counsel*"[All Fields] OR "ehealth*"[All Fields] OR "e health*"[All Fields] OR "einterv*"[All Fields] OR "e interv*"[All Fields] OR "etherap*"[All Fields] OR "e therap*"[All Fields] OR "mhealth*"[All Fields] OR "m health*"[All Fields] OR (("mobile"[All Fields] OR "mobiles"[All Fields]) AND "health*"[All Fields])) OR ("teleadvice"[All Fields] OR "teleassess*"[All Fields] OR "telecare"[All Fields] OR "teleconf*"[All Fields] OR "teleconsult*"[All Fields] OR "telecounsel*"[All Fields] OR "teledeliv*"[All Fields] OR "telehealth*"[All Fields] OR "teleinterv*"[All Fields] OR "telemanag*"[All Fields] OR "telemedic*"[All Fields] OR "telemonit*"[All Fields] OR "telenurs*"[All Fields] OR "telepharm*"[All Fields] OR "televisit*"[All Fields] OR "teletherapy*"[All Fields] OR "videochat*"[All Fields] OR "videotelephon*"[All Fields]) OR ("comput*"[Title/Abstract] OR "distance"[Title/Abstract] OR "electronic"[Title/Abstract] OR "internet"[Title/Abstract] OR "phone"[Title/Abstract] OR "online"[Title/Abstract] OR "remote"[Title/Abstract] OR "tele*"[Title/Abstract] OR "video"[Title/Abstract] OR "virtual"[Title/Abstract] OR "web"[Title/Abstract]) OR ("App"[Title/Abstract] OR "apps"[Title/Abstract] OR "facetime*"[Title/Abstract] OR "helpline*"[Title/Abstract] OR "store and forward*"[Title/Abstract] OR "store forward*"[Title/Abstract] OR "skype*"[Title/Abstract] OR "video*"[Title/Abstract] OR "zoom"[Title/Abstract] OR ("webbased"[All Fields] AND "tool"[Title/Abstract]) OR "web based tool*"[Title/Abstract] OR "voice-over"[Title/Abstract] OR "voiceover"[Title/Abstract] OR "VoIP"[Title/Abstract]) OR ("AI"[Title/Abstract] OR "artificial intelligence"[Title/Abstract] OR "machine-learning"[Title/Abstract] OR "DL"[Title/Abstract] OR "deep learning"[Title/Abstract] OR "DCNN"[Title/Abstract] OR "deep convolutional"[Title/Abstract]) OR ("artificial intelligence"[Title/Abstract] OR "machine-learning"[Title/Abstract] OR "machine-learning"[Title/Abstract] OR "machine learning algorithm"[Title/Abstract] OR "MLA"[Title/Abstract] OR "deep learning algorithm*"[Title/Abstract] OR "deep learning"[Title/Abstract] OR "deep neural network*"[Title/Abstract] OR "neural network*"[Title/Abstract] OR "machine-intelligence"[Title/Abstract] OR "machine-intelligence"[Title/Abstract])) AND 2010/01/01:3000/12/31[Date - Publication]

## **1b – List of HTA agencies included in the web search**

| **Country** | **Agency** | **Web link** |
| --- | --- | --- |
| Australia | Adelaide Health Technology Assessment (AHTA) | <https://www.adelaide.edu.au/ahta/> |
| **Europe:** |  |  |
| France | Haute Autoritè de Santè (HAS) | <https://www.has-sante.fr/> |
| Spain | Agency for Health Quality and Assessment of Catalonia (AQuAS) | <http://aquas.gencat.cat/ca/inici/> |
| UK | National Institute for Health and Care Excellence (NICE) | <https://www.nice.org.uk/> |
|  | NICE related – NHSX |  |
| Scotland | Healthcare Improvement Scotland (HIS) | <https://www.healthcareimprovementscotland.org/about_us/contact_healthcare_improvement.aspx> |
| Germany | Institut für Qualität und Wirtschaftlichkeit im Gesundheitswesen (IQWiG) | <https://www.iqwig.de/en/about-us/methods/results/hta-reports/> |
|  | Bundesinstitut für Arzneimittel und Medizinprodukte (BfArM) | <https://www.bfarm.de/DE/Home/_node.html> |
| Austria | The Austrian Institute for Health Technology Assessment (AIHTA) | <https://aihta.at/page/homepage/en> |
| Sweden | SWEDISH AGENCY FOR HEALTH TECHNOLOGY ASSESSMENT AND ASSESSMENT OF SOCIAL SERVICES (SBU) | <https://www.sbu.se/en/> |
| Finland | Finnish Institute for Health and Welfare (THL) | <https://thl.fi/en/web/thlfi-en> |
|  | Finnish Office of Health Technology Assessment (FinOHTA) | <http://www.stakes.fi/finohta/e/> |
|  | The Finnish Coordination Centre for Health Technology Assessment (FinCCHTA) | <https://www.ppshp.fi/Tutkimus-ja-opetus/FinCCHTA/Sivut/In_other_languages.aspx> |
| Italy | National Agency for Regional Health Services(AGENAS) | <https://www.agenas.gov.it/aree-tematiche/hta-health-technology-assessment> |
|  | Italian Society of Health Technology Assessment (Sihta) | <https://www.sihta.it/web/> |
|  | Italian Medicines Agency (AIFA) | <https://www.aifa.gov.it/en/web/guest/home> |
| Switzerland | Federal Office of Public Health FOPH (FPOH) | <https://www.bag.admin.ch/bag/de/home.html> |
| Belgium | Belgian Healthcare Knowledge center (KCE) | <https://kce.fgov.be/> |
| Denmark | Danish Health Authority (SST) | <https://www.sst.dk/en/english> |
|  | Centre for Innovative Medical Technology | <https://cimt.dk/gb/> |
|  | DEFACTUM | <https://www.defactum.dk/om-DEFACTUM/kerneydelser/medicinsk-teknologivurdering-mtv/> |
| Norway | The Norwegian Institute of Public Health | <https://www.fhi.no/en/qk/HTA/> |
| **America:** |  |  |
| Canada | Canadian Coordinating Office for Health Technology Assessment(CADTH) | <https://cadth.ca/> |
|  | The Institut national d’excellence en santé et en services sociaux (INESSS) | <https://www.inesss.qc.ca/en/home.html> |
| Brazil | National Committee for Health Technology Incorporation (CONITEC) | <http://www.conitec.gov.br/> |
| US | Food and Drug Administration (FDA) | <https://www.fda.gov/> |
|  | FDA - Digital Health Center of Excellence | <https://www.fda.gov/medical-devices/digital-health-center-excellence> |
|  | Institute for Clinical and Economic Review (ICER) | <https://icer.org/> |

## **Appendix 1c – List of the panel of experts for the Delphi study**

In alphabetic order:

| **Title** | **Name** | **Surname** | **Country** |
| --- | --- | --- | --- |
| Dr. | Oda | Bakken | Norway |
| Prof. | Stefania | Boccia | Italy |
| Dr. | Randi | Borgen | Norway |
| Dr. | Laura | Burkhardt | Germany |
| Prof. | Federico | Cabetza | Italy |
| Prof. | Americo | Cicchetti | Italia |
| Prof. | Andrea | Damiani | Italy |
| Dr. | Signe | Daugbjerg | Italy |
| Dr. | Rossella | Di Bidino | Italy |
| Dr. | Bernice | Dillon | United Kingdom |
| Prof. | Isabelle | Durand-Zaleski | France |
| Dr. | Iben | Fasterholdt | Denmark |
| Prof. | Brynjar | Fure | Sweden |
| Prof. | Chris | Gastmans | Belgium |
| Dr. | Rachel | Giles | Netherlands |
| Dr. | Dianne | Gove | Luxembourg |
| Dr. | Tim | Govers | The Netherlands |
| Dr. | Pooyeh | Graili | Canada |
| Prof. | Wolfgang | Greiner | Germany |
| Dr. | Christoffer | Hatlestad-Hall | Norway |
| Prof. | Ira R J | Hebold Haraldsen | Norway |
| Dr. | Mouna | Jameleddine | Tunisia |
| Dr. | Kristian | Lampe | Finland |
| Dr. | Alessandra | Lo Scalzo | Italy |
| Dr. | Marco | Marchetti | Italy |
| Prof. | Camillo | Marra | Italy |
| Dr. | Frida | Milella | Italy |
| Dr. | Jean | Mossman | United Kingdom |
| Dr. | Anne | Oeksengaard | Norway |
| Prof. | Monica | Oliveira | Portugal |
| Prof. | Wija | Oortwijn | Netherlands |
| Prof. | Luis | Pena | Spain |
| Dr. | Elena | Petelos | Greece |
| Prof. | Hanna | Renvall | Finland |
| Prof. | Walter | Ricciardi | Italy |
| Prof. | Dario | Sacchini | Italia |
| Dr. | Alejandro | Sanchez-Rico | Spain |
| Dr. | Jacopo | Scipione | Italy |
| Dr. | Ann | Single | Australia |
| Prof. | Sonia | Sousa | Estonia |
| Prof. | Panos | Stafylas | Greece |
| Prof. | Vincenzo | Valentini | Italy |
| Dr. | Kicky | van Leeuwen | Netherlands |
| Dr. | Tijs | Vandemeulebroucke | Germany |
| Prof. | Anis | Yazidi | Norway |
