# Supplementary material for: Health technology assessment framework for artificial intelligence-based technologies
Source: Int J Technol Assess Health Care. 2024 Nov 21;40(1):e61. doi: 10.1017/S0266462324000308 (PMC11703629; doi:10.1017/S0266462324000308)
Supplement: Di Bidino et al. supplementary material 2 — Di Bidino et al. supplementary material [file S0266462324000308sup002.docx]

## **Supplementary Material 2**

## **List of HTA topics included**

| Domains | HTA issues | Additional info |
| --- | --- | --- |
| Domain 1: Health Problem and Current Use of the Technology | Target condition | Health problem and targeted population, burden and symptoms of disease, risk factors, areas of condition targeted by the technology |
|  | Current management of the condition | Current treatment/diagnosis, guidelines, alternative treatments |
|  | Utilization | Health condition, population and purpose for  technology use, the spread of use of technology, variation of use, innovation level of technology |
| Domain 2: Description and technical characteristics of Technology | Features of the technology | Phase of development and implementation, benefit in relation to comparators, context of use, reference values |
|  | Regulatory Status | CE marking, reimbursement status of the technology |
|  | Investments and tools required to use the Technology | Special material investments, equipment or  premises needed; data/records and/or registry needed to monitor use |
|  | Training and information needed to use the technology | Requirements and qualifications needed to use technology (for professionals/ caregivers/ patients) |
| Domain 3: Safety | Patient Safety | False positive, false negative and incidental findings, susceptible patient groups, change of harm over time and in different settings |
|  | Occupational safety | Possible occupational harms |
|  | Environmental safety | Risks for the public and the environment |
|  | Risk managment | Safety risks for patients, professionals and environment, kind of data/records and/or registry needed to monitor use |
| Domain 4: Clinical Effectiveness | Mortality & morbidity | New technology’s effect on mortality, disease severity, magnitude & frequency of morbidity, disease progression & recurrence) |
|  | Function | Effect on body function and work ability, effect on daily lives and return to previous state |
|  | Health-related quality of life & Quality of life | Generic healthrelated/ disease-specific/non-healthrelated quality of life |
|  | Patient Satisfaction & Patient safety | Patient satisfaction, consequences of false positive, false negative and incidental findings |
|  | Test-treatment chain and accuracy | Available treatment for detected condition, accuracy compared to optional tests, reference standard, requirement of accuracy, test variation, test reliably |
|  | Change-in-Management | Improved detection, change in management decisions, need for hospitalisation |
|  | Benefit-harm balance | Overall benefits and harms of the technology  in health outcome |
| Domain 5: Costs and economic evaluation | Resource utilisation | Identification and measurement of resources used (new technology and comparator), measured and/or estimated costs of the new technology and its comparator(s), budget impacts, modifications of resource need of other technologies |
|  | Measurement and estimation of outcomes | Outcome identification, measurement and  valuation |
|  | Examination of costs and outcomes | Differences in costs and outcomes |
|  | Characterising uncertainty | Uncertainties in cost and economic evaluation |
|  | Characterising heterogeneity | Differences in outcomes & cost effectiveness explained by subgroup variation |
|  | Validity of the model(s) | Methodological assumptions, validity of economic evaluation estimates |
| Domain 6: Ethical analysis | Benefit-harm balance | Symptoms, burden of disease or health condition (patient), benefits & harms for others, hidden or unintended consequences, ethical obstacles for evidence generation |
|  | Autonomy | Targeted vulnerable patients, effect on patient ́s capability, need for supportive action  for information autonomy, challenges related to implementation or withdrawal of technology) |
|  | Respect for persons | Human dignity, moral, religious or cultural  integrity, sphere of privacy |
|  | Justice and Equity | Effect on distribution of health care resources,  factors affecting access to technology |
|  | Legislation | Basic human rights, new ethical challenges |
|  | Ethical consequences of the HTA | Ethical consequences of endpoints, cut-off values and comparators/controls, data & assumptions, technology assessment |
| Domain 7: Organisational aspects | Health delivery process | Current work processes, patient flow, proper training of staff, involvement of patients/caregivers, co-operation of activities, quality assurance and monitoring of new technology |
|  | Structure of health care system | Distribution requirements and implementation, ensuring access to new technology |
|  | Process-related costs | Acquisition & set up, modification of need & resource use, implementation budget impact |
|  | Management | Management problems & opportunities in relation to technology, basis for eligibility of  use of technology |
|  | Culture | Other group interests in planning/ implementation, technology acceptance |
| Domain 8: Patients and Social aspects | Patients’ perspectives | Experience of living with the condition, expectation of technology and use, burden of care-givers |
|  | Social group aspects | Current access to therapies, factors that prevent access to technology |
|  | Communication aspects | Explanation of treatment choices, specific communications to improve adherence |
| Domain 9: Legal aspects | Autonomy of the patient | Legal requirements for information provision, consent of minors and incompetent persons |
|  | Privacy of the patient | Information violating privacy, laws about sharing results, laws for securing patient data |
|  | Equality in health care | Laws/binding rules for access, EU-level and national regulations in equal access to technology |
|  | Ethical aspects | Effect on basic human rights, new ethical challenges |
|  | Authorisation and safety | Authorisations and register listings, laws/binding rules requirement in terms of safety |
|  | Ownership and liability | Intellectual property rights and license fees, manufacturers guarantee rules |
|  | Regulation of the market | Conflicts of interest, new legal issues, marketing |
| Additional topics | Appropriateness | Is the technology necessary and appropiate in each clinical setting, is there a real-world benefit of the technology? |
|  | Trustworthiness | Follows the requirements of Trustworthy AI. e.g. how lawful, ethical, robust is the AI |
|  | Human agency and oversight | Human-in-the-loop, inclusion of feedback loop, capability of technology to interact and work with humans, management by humans |
|  | Patient centeredness | Shared decision making, inclusion of individual preferences |
|  | Interpretability | The extent to which a cause and effect can be  observed within the AI system |
|  | Explainability | Ability to explain both the technical processes and the reasoning, behind the decisions or predictions of the AI system in human terms |
|  | Social sustainability | Impacts, both positive and negative, on people and human rights |
|  | Environmental sustainability | Assessment of carbon footprint of the technology |
|  | Accountability and compensation | Processes of procedures to insure accountability for adverse events caused by the technology, procedures for the compensation of undue damage |
|  | Bias in data | Data inclusiveness, diversity, non discrimation, fairness |
|  | Bias in the development phase of algorithms | Disclosure in use of proxies, inclusiveness, diversity, non discrimation, fairness |
|  | Accuracy of AI model | How closely an AI system’s predictions match the truth |
|  | Individual control of data | Willingness of individuals to share health data |
|  | Data protection | Protection of personal data relating to individuals in line with the General Data Protection Regulation (GDPR) |
|  | Data acquisition and use | Separation between training, tuning, and test datasets |
|  | Learning and training of models | Retraining strategy of model, organization of learning and control phases, representativeness of data for training and labelling purpose |
|  | Periodic evaluation | According to new available scientific knowledge and according to algorithm development |
|  | Discloser of limitations | Of technology use (e.g. only for specific populations groups or equipment) |
|  | Cross vendor compatibility | Level of cross vendor availability exists for the current technology |
|  | Manufacture of the technology | Descriprion of process of creation/production of the alghoritm and software |
