# Supplementary material for: Health technology assessment framework for artificial intelligence-based technologies
Source: Int J Technol Assess Health Care. 2024 Nov 21;40(1):e61. doi: 10.1017/S0266462324000308 (PMC11703629; doi:10.1017/S0266462324000308)
Supplement: Di Bidino et al. supplementary material 3 — Di Bidino et al. supplementary material [file S0266462324000308sup003.docx]

## **Supplementary Material 3**

## **Topic level responses**

|  | **N** | | | **% on total responses** | | |
| --- | --- | --- | --- | --- | --- | --- |
|  | Critical | Important | No important | Critical | Important | No important |
| **Health Problem and Current Use of the Technology** |  |  |  |  |  |  |
| Target condition | 43 | 3 | 0 | **93.48%** | 6.52% | 0.00% |
| Current management of the condition | 38 | 7 | 0 | **84.44%** | 15.56% | 0.00% |
| Utilization | 35 | 10 | 0 | **77.78%** | 22.22% | 0.00% |
| **Description and technical characteristics of technology** |  |  |  |  |  |  |
| Features of the technology | 43 | 2 | 1 | **93.48%** | 4.35% | 2.17% |
| Regulatory Status | 33 | 12 | 1 | **71.74%** | 26.09% | 2.17% |
| Investments and tools required to use the Technology | 34 | 10 | 0 | **77.27%** | 22.73% | 0.00% |
| Training and information needed to use the technology | 39 | 7 | 0 | **84.78%** | 15.22% | 0.00% |
| **Safety** |  |  |  |  |  |  |
| Patient Safety | 44 | 1 | 1 | **95.65%** | 2.17% | 2.17% |
| Occupational safety | 29 | 13 | 2 | 65.91% | 29.55% | 4.55% |
| Environmental safety | 26 | 15 | 4 | 57.78% | 33.33% | 8.89% |
| Risk managment | 43 | 3 | 0 | **93.48%** | 6.52% | 0.00% |
| **Clinical Effectiveness** |  |  |  |  |  |  |
| Mortality & morbidity | 38 | 5 | 0 | **88.37%** | 11.63% | 0.00% |
| Function | 38 | 2 | 0 | **95.00%** | 5.00% | 0.00% |
| Health-related quality of life & Quality of life | 40 | 5 | 0 | **88.89%** | 11.11% | 0.00% |
| Patient Satisfaction & Patient safety | 40 | 6 | 0 | **86.96%** | 13.04% | 0.00% |
| Test-treatment chain and accuracy | 38 | 8 | 0 | **82.61%** | 17.39% | 0.00% |
| Change-in-Management | 30 | 14 | 1 | 66.67% | 31.11% | 2.22% |
| Benefit-harm balance | 43 | 2 | 1 | **93.48%** | 4.35% | 2.17% |
| **Costs and economic evaluation** |  |  |  |  |  |  |
| Resource utilisation | 38 | 6 | 1 | **84.44%** | 13.33% | 2.22% |
| Measurement and estimation of outcomes | 41 | 3 | 1 | **91.11%** | 6.67% | 2.22% |
| Examination of costs and outcomes | 36 | 7 | 1 | **81.82%** | 15.91% | 2.27% |
| Characterising uncertainty | 31 | 11 | 2 | **70.45%** | 25.00% | 4.55% |
| Characterising heterogeneity | 32 | 12 | 1 | **71.11%** | 26.67% | 2.22% |
| Validity of the model(s) | 35 | 8 | 2 | **77.78%** | 17.78% | 4.44% |
| **Ethical analysis** |  |  |  |  |  |  |
| Benefit-harm balance | 43 | 2 | 0 | **95.56%** | 4.44% | 0.00% |
| Autonomy | 39 | 5 | 0 | **88.64%** | 11.36% | 0.00% |
| Respect for persons | 40 | 5 | 0 | **88.89%** | 11.11% | 0.00% |
| Justice and Equity | 37 | 7 | 0 | **84.09%** | 15.91% | 0.00% |
| Legislation | 37 | 7 | 1 | **82.22%** | 15.56% | 2.22% |
| Ethical consequences of the HTA | 35 | 8 | 3 | **76.09%** | 17.39% | 6.52% |
| **Organisational aspects** |  |  |  |  |  |  |
| Health delivery process | 31 | 13 | 2 | 67.39% | 28.26% | 4.35% |
| Structure of health care system | 29 | 17 | 0 | 63.04% | 36.96% | 0.00% |
| Process-related costs | 34 | 10 | 1 | **75.56%** | 22.22% | 2.22% |
| Management | 25 | 18 | 2 | 55.56% | 40.00% | 4.44% |
| Culture | 22 | 21 | 1 | 50.00% | 47.73% | 2.27% |
| **Patients and Social aspects** |  |  |  |  |  |  |
| Patients’ perspectives | 37 | 8 | 0 | **82.22%** | 17.78% | 0.00% |
| Social group aspects s | 24 | 19 | 1 | 54.55% | 43.18% | 2.27% |
| Communication aspects | 26 | 16 | 2 | 59.09% | 36.36% | 4.55% |
| **Legal aspects** |  |  |  |  |  |  |
| Autonomy of the patient | 39 | 6 | 0 | **86.67%** | 13.33% | 0.00% |
| Privacy of the patient | 40 | 5 | 0 | **88.89%** | 11.11% | 0.00% |
| Equality in health care | 34 | 11 | 0 | **75.56%** | 24.44% | 0.00% |
| Ethical aspects | 33 | 9 | 0 | **78.57%** | 21.43% | 0.00% |
| Authorisation and safety | 31 | 8 | 4 | **72.09%** | 18.60% | 9.30% |
| Ownership and liability | 27 | 12 | 4 | 62.79% | 27.91% | 9.30% |
| Regulation of the market | 22 | 17 | 2 | 53.66% | 41.46% | 4.88% |
| **Additional topics** |  |  |  |  |  |  |
| Appropriateness | 40 | 4 | 2 | **86.96%** | 8.70% | 4.35% |
| Trustworthiness | 38 | 6 | 1 | **84.44%** | 13.33% | 2.22% |
| Human agency and oversight | 39 | 6 | 0 | **86.67%** | 13.33% | 0.00% |
| Patient centeredness | 28 | 14 | 1 | 65.12% | 32.56% | 2.33% |
| Interpretability | 31 | 12 | 1 | **70.45%** | 27.27% | 2.27% |
| Explainability | 34 | 7 | 3 | **77.27%** | 15.91% | 6.82% |
| Social sustainability | 23 | 19 | 0 | 54.76% | 45.24% | 0.00% |
| Environmental sustainability | 22 | 16 | 5 | 51.16% | 37.21% | 11.63% |
| Accountability and compensation | 31 | 11 | 1 | **72.09%** | 25.58% | 2.33% |
| Bias in data | 42 | 4 | 0 | **91.30%** | 8.70% | 0.00% |
| Bias in the development phase of algorithms | 36 | 9 | 1 | **78.26%** | 19.57% | 2.17% |
| Accuracy of AI model | 44 | 1 | 0 | **97.78%** | 2.22% | 0.00% |
| Individual control of data | 33 | 7 | 4 | **75.00%** | 15.91% | 9.09% |
| Data protection | 39 | 5 | 2 | **84.78%** | 10.87% | 4.35% |
| Data acquisition and use | 34 | 6 | 3 | **79.07%** | 13.95% | 6.98% |
| Learning and training of models | 32 | 9 | 1 | **76.19%** | 21.43% | 2.38% |
| Periodic evaluation | 30 | 13 | 1 | 68.18% | 29.55% | 2.27% |
| Discloser of limitations of technology use | 37 | 5 | 0 | **88.10%** | 11.90% | 0.00% |
| Cross vendor compatibility | 14 | 21 | 5 | 35.00% | 52.50% | 12.50% |
| Manufacturer/developer of the technology | 15 | 23 | 7 | 33.33% | 51.11% | 15.56% |
